# Solidarity, support and acceptance: a celebration of the person, not the disease

**DOI:** 10.1007/s00520-022-06932-8

**Published:** 2022-02-27

**Authors:** Tamara Page

**Affiliations:** University of Adelaide & St Andrew’s Hospital, Adelaide, SA Australia

**Keywords:** Support, Acceptance, Body image, Resilience, Sharing, Solidarity

## Abstract

St Andrew’s Hospital provides care for more than 700 patients diagnosed with breast cancer annually. Each person’s experience is individual, but created by their interactions with the healthcare providers from diagnosis through to treatment. The ability of St Andrew’s Hospital to deliver this care and create the best possible outcomes is crucial to each person’s recovery. Those affected by breast cancer and having undergone treatment at St Andrew’s Hospital are invited to an annual luncheon where guest speakers provide powerful presentations that align with each person’s personal journey post-treatment, providing them the ability to reflect on what bought them here, and how to plan moving forward. A number of exhibitors offering both free and for purchase items or services are available for people to look at, book into or purchase. The highlight of the day is the lingerie fashion parade where women gather the courage to display custom-made lingerie and swimwear, enabling women post-mastectomy to celebrate themselves and not the disease.

Solidarity, support and acceptance, words used to describe the benefit of an annual celebration held by St Andrew’s Hospital for people who have been diagnosed with breast cancer. Breast cancer is a defining moment in anyone’s life; from that first call or text message following your annual mammogram to the diagnosis and subsequent treatment. The importance of a breast care team to provide the emotional, physical and psychological support is paramount during this time. St Andrew’s Hospital is a stand-alone private hospital that hosts a multidisciplinary breast clinic service providing specialist knowledge in the fields of breast imaging, diagnosis and treatment — all within one private area.

Breast Cancer Network Australia (BCNA) [1] state it is often surprising for many to learn that breast cancer does not discern between males and females and that whilst it is rare (less than 1% of all cancers in men), it is a disease that impacts approximately 150 men and 20,000 women annually [2].

The Breast care team at St Andrew’s Hospital see more than 700 patients annually and it is primarily nurses who accompany and support the patient following their treatment. Some patients require a mastectomy and others may choose less radical procedures such as breast-conserving surgery. In a systematic review by Martins et al. [3], it was identified that mastectomy worsened women’s body image, sexual functioning and quality of life in comparison to breast-conserving surgery. No matter what treatment option the patient chooses, the importance of promoting ways to support patients through their illness in order to improve their quality of life is paramount [4].

A Breast Care Nurse (BCN) at St Andrews Hospital recognised the importance of supporting patients through their illness and in 1998, the inaugural Annual Breast Cancer Day was held. An inspiring event that allowed patients to network and gather information to assist in their ongoing recovery initially started as a morning tea on the grounds of the hospital, facilitated by the BCNs for the patients they had cared for and has now grown into a gala event with approximately 250 people attending. This event provided by the Breast care team is a great opportunity for people who have recently gone through breast cancer treatment to meet other people going through similar experiences. Each day of their admission, patients are seen by a BCN and prior to discharge are provided with written material which includes written information and an Annual Breast Cancer Day pre-invite.

All patients in the previous 2 years are invited to the free event and are able to bring one support person with them for a nominal fee. The event has guest speakers, exhibitors and entertainment acts to bring together an uplifting day of laughter, education and fun. The BCNs undertake most of this event in their own time.

The importance of this event was highlighted in an online forum from the BCNA page where a number of people identified the value of the annual event [5].

I went to one of their luncheons and really enjoyed it. It was a great opportunity to meet other women going through similar experiences. It was what I needed at the time, solidarity, support and acceptance.

GREAT NEWS!! I will be going & can’t wait to catch up with you & anyone else. …...how wonderful it will be & quite emotional having been “forum buddies” for so long & finally getting to hug you all in person!! I’m soooooo excited now!!!!

Hope you doing OK & recovering after the radio. It does still take a while after treatment finishes so don’t push yourself as you have to be fit for the luncheon next week!!

What a special day this was. the first cancer function I have been to & it certainly didn’t disappoint. Many words & emotions come to mind when I think back on the day - Support, Love, sharing but probably the word I would best use is EMPOWERING!! the speakers were amazing but the highlight of the day was meeting my very first forum buddie. This amazing woman is even more beautiful in the flesh, both inside & out & it was quite emotional & overwhelming to finally meet someone you have grown to know, in person. We both first posted at the same time, were both diagnosed at the same time, surgery together at the same hospital…

The comments in the online forum identify the supportive nature of the event as well as people having ongoing support from others going through a similar experience. During an interview conducted on 8th December, 2021, Derek McManus a previous guest speaker spoke of five important journey pillars: optimism, influence, passion or purpose, planning and support and agreed that you could feel the emotion and optimism from the crowd.

It is a positive event for not only the patients who provide feedback on the day and talk about how they find it an encouraging part of their breast cancer journey, but also for the BCN team who gain much pleasure from seeing past patients and enjoy touching base with them again.

Alongside of the powerful presentations from the guest speakers and the range of exhibitors the big highlight of the event is the Lingerie and Swimwear Fashion Parade. The men folk are asked to adjourn to another room and several women who have previously undergone breast cancer surgery take part in the fashion parade. Garments are provided from a local company that specialises in post-mastectomy garments. Women who have lost a breast may regard themselves as disfigured resulting in a discrepancy between their self and the societal image of a woman [6]; so for many of these women to gather the courage to parade in lingerie and swimwear in front of others demonstrates a solidarity and acceptance from others that is all empowering and ‘proves that you can look and feel feminine and fabulous after breast cancer surgery’ [7].

## Conclusion

St Andrew’s Hospital breast care team supports more than 700 patients annually through their breast cancer journey and whilst the anecdotal data provided supports the idea that the Annual Breast Cancer Day provides solidarity, support and acceptance, the true value of an event such as this on the patient’s body image perception and quality of life necessitates further research.
